# Supplementary material for: Sugar Beet Pulp as a Biorefinery Substrate for Designing Feed
Source: Molecules. 2023 Feb 22;28(5):2064. doi: 10.3390/molecules28052064 (PMC10004680; doi:10.3390/molecules28052064)
Supplement: Supplementary file 1 [file molecules-28-02064-s001.zip › molecules-2208885-supplementary.pdf]

Supplementary Material

Sugar Beet Pulp as a Biorefinery Substrate for Designing Feed

Dawid Dygas *, Dorota Kręgiel *, Joanna Berłowska *

Department of Environmental Biotechnology, Lodz University of Technology, 171/173 Wólczańska Street, 90-530 Łódź, Poland

Table S1. Calculated enzyme units [U].

Enzyme preparation	Dose	Cellulase	Invertase	Xylanase	Pectinase
Viscozyme	0.5	10.5	30.6	13.0	156.3
	0.25	5.2	15.3	6.5	78.2
	0.125	2.6	7.7	3.2	39.1
Ultraflo Max	0.5	16.4	0.9	32.4	10.6
	0.25	8.2	0.5	16.2	5.3
	0.125	4.1	0.2	8.1	2.7
